# Secondary Mitochondrial Dysfunction in Gaucher Disease Type I, II and III—Review of the Experimental and Clinical Evidence

**DOI:** 10.3390/genes16111269

**Published:** 2025-10-28

**Authors:** Mollie Dewsbury, Tyler Purcell, Derralynn Hughes, Aimee Donald, Iain P. Hargreaves, Karolina M. Stepien

**Affiliations:** 1 School of Pharmacy and Biomolecular Sciences, Liverpool John Moores University, Liverpool L3 3AF, UK; m.r.dewsbury@2024.ljmu.ac.uk (M.D.); tyler.purcell@postgrad.manchester.ac.uk (T.P.); i.p.hargreaves@ljmu.ac.uk (I.P.H.); 2 Royal Free London NHS Foundation Trust, University College London, Pond Street, London NW3 2QG, UK; derralynnhughes@nhs.net; 3 Manchester Foundation Trust, Oxford Road, Manchester M13 9WL, UK; aimee.donald@manchester.ac.uk; 4 Neurometabolic Unit, National Hospital, London WC1N 3BG, UK; 5 Salford Royal Hospital, Northern Care Alliance NHS Foundation Trust, Salford M6 8HD, UK; 6 Division of Cardiovascular Sciences, University of Manchester, Oxford Road, Manchester M13 9WL, UK

**Keywords:** Gaucher disease, oxidative stress, secondary mitochondrial dysfunction

## Abstract

Gaucher disease (GD) is an autosomal recessive metabolic disorder caused by pathogenic variants in the *GBA1* gene, which encodes the lysosomal hydrolase β-glucocerebrosidase (GCase). The pathogenic defects result in a misfolded protein, which can trigger endoplasmic reticulum stress and an unfolded protein response within the affected cells. The reduced enzyme activity leads to accumulation of its substrates, glucosylceramide (GlcCer) and glucosylsphingosine (GlcSph), within lysosomes or macrophages and with prominent disease manifestations in reticuloendothelial tissues such as liver, spleen and bone marrow. GCase defects alter both the mitochondria and the lysosome. In the lysosome, reduced GCase activity leads to glycosphingolipid build-up, disrupting lysosomal function and autophagy, thereby activating α-synuclein accumulation. GCase can also be imported into the mitochondria, where it fosters the integrity and function of mitochondrial respiratory chain (MRC) complex I. Thus, the reduced GCase activity impairs the normal mitochondrial function and increases oxidative stress in this organelle, which may contribute to cell death. However, further studies are required to confirm this mechanism of MRC dysfunction. In this review we have systematically evaluated the evidence for oxidative stress in individuals affected by GD, as well as the currently available therapies and adjunctive therapies. Therapies targeting oxidative stress may prove useful as adjuvant treatments for GD.

## 1. Introduction

**Figure 1:**
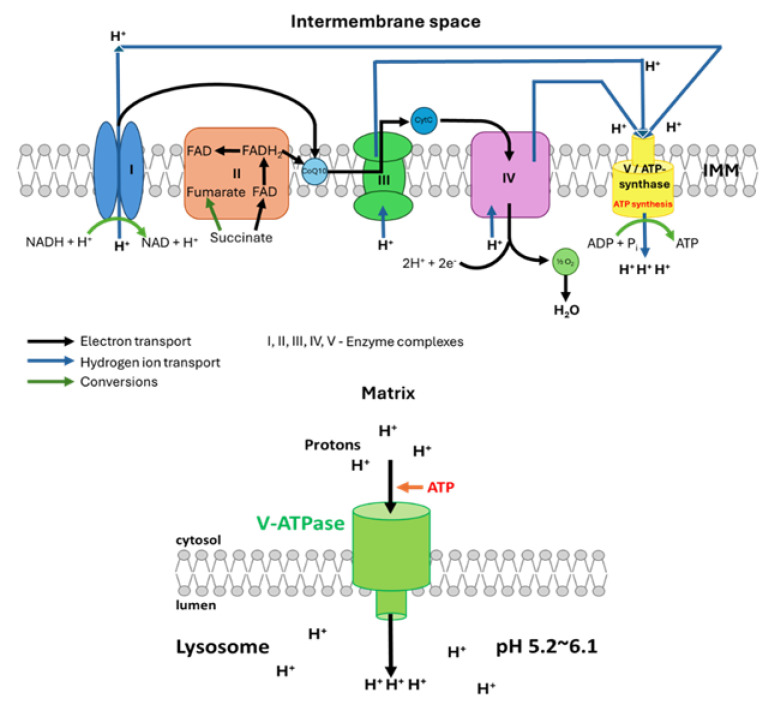
The mitochondrial respiratory chain (MRC) complexes I to IV and the function of V-ATPase within the lysosomal membrane. The MRC generates ATP via the exchange of protons along the electron transport chain (ETC). The ATP produced is utilised by the V-ATPase enzyme present within the lysosomal membrane for the active transport of protons from the cytosol to the lysosomal lumen. This is necessary to maintain the acidic lysosomal pH.

Lysosomal storage disorders (LSDs) are a heterogeneous group of rare inherited metabolic diseases, caused by the deficiency or defective function of lysosomal enzymes or transporters. This leads to the gradual accumulation of undigested substances within the lysosome. LSDs are individually rare, but collectively they are a prevalent group of metabolic disorders, affecting approximately 1 in 5000 live births [1]. Gaucher disease (GD), especially type I in Europe and the US (GD I; OMIM# 23080, 231000, 231005), is one of the most commonly occurring LSDs [2]. GD can be divided into three clinical subtypes. GD1 generally has no central nervous system (CNS) involvement, whereas types II and III are neuronopathic forms of the disease [2] (Table 1). This autosomal recessive disorder is caused by a pathogenic variant in the *GBA* gene [3], leading to the deficiency of GCase, a lysosomal hydrolase [3]. This results in the accumulation of its substrate, glucocerebroside, within the lysosome, thereby compromising cellular function [4]. Other organelles can also be affected, including the mitochondria. The secondary alteration in mitochondrial function within LSDs has been indicated by morphological changes within the mitochondria, a reduction in mitochondrial membrane potential, reduced ATP generation and an increase in the production of reactive oxygen species (ROS) [5]. The mitochondria are essential for normal lysosomal function, which has been shown to be diminished under conditions with dysfunctional mitochondria, reducing their activity and lysosomal acidification [6]. The production of ATP in the mitochondria is essential for the V-ATPase enzyme in the lysosomal membrane to utilise its free energy to pump protons into the lysosomal lumen to maintain its acidity (Figure 1). Therefore, if this process is diminished due to dysfunctional mitochondria, the function of lysosomal hydrolases will be reduced as a result of a sub-optimal acidic environment (pH 5.2–6.1) [7].

Neurological dysfunction is a prominent clinical manifestation in both LSDs and primary mitochondrial disease, although the pathophysiological causes of this impairment have yet to be fully elucidated in these disorders. Lysosomes carry out the cellular process of the degradation and recycling of macromolecules and organelles, known as autophagy [16]. The autophagic degradation of mitochondria, known as mitophagy, is also performed by the lysosome, allowing for the removal of defective and/or dysfunctional mitochondria from the cell. This process is essential in cells with damaged mitochondria to prevent the accumulation of ROS, but this may be compromised in the presence of defective lysosomal function. This results in the accumulation of dysfunctional mitochondria, further compromising cellular energy status [17]. The presence of either organelle being dysfunctional appears to affect the other, and in turn, both organelles become negatively affected. The maintenance of lysosomal structure and function by mitochondrial activity has been demonstrated in several studies. Specifically, disrupting the function of the mitochondria diminishes lysosomal acidification and activity, as well as causing the accumulation of enlarged endo-lysosomal structures [6]. Following the inhibition of mitochondria via deletion of mitochondrial proteins AIF, OPA1 or PINK1, in addition to the inhibition of the mitochondrial respiratory chain (MRC), lysosomal activity is impaired and cells present with large lysosomal vacuoles [18].

This review discusses the potential causes of secondary mitochondrial dysfunction in GD, outlining the evidence from in vitro, animal and clinical investigations. This review also discusses the mechanisms of action of currently available therapies together with potential adjunct therapies to target mitochondrial dysfunction in GD. However, the latter therapies will require thorough clinical investigations before they can be used in GD.

## 2. Methods

A comprehensive literature review was conducted of reports from the Pubmed, Embase and CINAHL databases, as well as those found by manually entering the same key terms separately. Applied filters were Human Species and English Language, and the research timeframe spanned from January 1970 to August 2025.

Original studies, reviews, case reports and case series including cases, clinical trials and experimental studies on GD and evidence of mitochondrial dysfunction, oxidative stress and neurodegeneration were included. Data from abstracts of which the full text could not be found, or which were not written in English, were excluded. The data extraction was performed from each full text by the authors (M.D., K.M.S. and I.P.H).

The search was performed using combinations of several keywords: ‘Gaucher disease’, ‘oxidative stress’, ‘antioxidants’, ‘vitamin E’, ‘tocopherol’, ‘clinical trials’, ‘neurodegeneration’, ‘lysosomal storage disease’, ‘central nervous system’, ‘ROS’, ‘mitochondrial’, ‘mitochondrial dysfunction’, ‘clinical outcome’ and ‘mortality’, with Boolean operators “AND” and “OR” for a thorough search. We performed a descriptive narrative synthesis. The outcome of the literature search and the search strategies are in the Supplementary Materials.

## 3. Neurological Manifestations and GD

Besides neurological impairment, there is a broad spectrum of clinical manifestations that present in GD, and the severity of these presentations is dependent on the relative form of the disease. In addition to the central nervous system (CNS), haematological, visceral and bone systems are all affected due to the accumulation of glucocerebroside in tissue macrophages [19]. Consequently, patients present with cytopenia, hepatosplenomegaly and bone lesions, as Gaucher cells infiltrate the bone marrow, spleen and liver [20]. However, these symptoms are highly variable amongst patients with GD, where symptoms, age of onset, severity and rate of progression are heterogenous.

Type 2 and 3 GD are both neuronopathic, causing severe neurological symptoms including seizures, spasticity and cognitive decline [21], but there is some variation in presentation and severity between the two types (Table 1). Although type 1 is considered to be non-neuronopathic, some of these patients and carriers have shown increased risk of Parkinsonism and dementia [22]. The *GBA1* gene presented in the heterozygous status is the most common genetic risk factor for Parkinson’s disease (PD) [23,24]. This was demonstrated in a study from Sidransky et al. (2009) [25], where patients who carried one of the *GBA1* pathogenic variants presented much earlier with PD and were at an increased risk of having atypical clinical manifestations and having relatives that were more likely to be affected, compared to patients who did not carry the *GBA1* variant. *GBA1*-associated PD (*GBA1*-PD) is therefore being increasingly recognised as its own distinct entity within the Parkinsonism disorder spectrum [26]. Typically, *GBA1*-PD age of onset is much earlier and has a more aggressive progression than iPD (idiopathic PD). There is also a generally reduced response to generic PD medication [26]. *GBA1*-PD is characterised by pronounced cognitive impairment and increased non-motor symptoms compared to iPD. Patients with *GBA1*-PD also tend to exhibit Lewy bodies more diffusely throughout the brain, exacerbating neurodegenerative damage [26]. Given the increased risk of PD in these patients, it is crucial to screen PD patients for underlying GD, as the latter can be effectively treated [26].

### Pathophysiology of Neurological Complications in GD

Pathogenic variants in the *GBA1* gene lead to two major outcomes: (i) the production of an aberrant GCase, which may be partially retained in the ER or removed through autophagy mechanisms and (ii) substrates (i.e., GlcCer and GlcSph) accumulation [27,28].

In GD, GlcSph accumulates in addition to GlcCer. A study has shown that GCase activity is lower and that GLcSph levels are elevated in *GBA1*-PD patients relative to *GBA1* controls, irrespective of the underlying *GBA1* variant [29]. Therefore, the increased levels of GlcSph in PD could serve as a potential biomarker for the disease and warrant further investigation. GCase expression in the brain is abundant, particularly within dopaminergic neurons [30,31]. As a result, dysfunctional GCase may interfere with their normal function. Reduced activity of GCase mediates the onset of PD in these patients by causing increased aggregation of α-synuclein, a protein that plays a role in regulating the trafficking of synaptic vesicles and the release of neurotransmitters [32]. Reduced GCasea activity may cause autophagy-lysosomal dysfunction, inducing endoplasmic reticulum (ER) stress, mitochondrial damage and inflammatory responses, as well as affecting lipid metabolism. It has been recognised that ER stress and the dysfunctional unfolded protein response are significant contributors to neurological symptoms in various diseases [33]. The impaired activity and misfolding of GCase proteins cause them to become retained in the ER, which in turn promotes ER stress [33]. The levels of increased ER stress and activation of the unfolded protein leading to apoptosis in neuronal cells varies. The presence of modifying genes is a potential explanation for this [21,34]. The unfolded proteins are retrotranslocated from the ER to the cytosol, where they are eliminated by the ubiquitin–proteasome system, through the ER-associated degradation (ERAD) process. The mutant proteins are also identified as misfolded and therefore are retained in the ER [35].

The lack of functional GCase within the lysosome results in an impaired autophagic lysosomal pathway (ALP), leading to the accumulation of fat-containing substrates including GlcCer, as well as aggregation of α-synuclein [35,36]. This accumulation stops GCase trafficking from the ER/Golgi to lysosomes, worsening lysosomal dysfunction. Deficient GCase and impaired ALP activity then impact mitochondrial function, leading to increased ROS, reduced ATP production and distorted mitochondrial structure [37]. GCase loss combined with lipid accumulation and α-synuclein aggregation can also initiate microglial activation and neuroinflammation. Collectively, these factors contribute to neuronal cell death and the development of PD [23]. *GBA1*-PD differs from idiopathic PD (iPD) in its pathophysiological features as well as its clinical progression and presentation. A 2024 study by Ducatez et al. [38] utilised immune-based proteomics and mass spectrometry-based metabolomics techniques to demonstrate increased phosphatidylcholine levels in GD patients, suggesting disruptions in lipid metabolism, likely due to enhanced synthesis. This points to ER stress and impaired lipid trafficking, which are common features of LSDs. The patients also displayed an inflammatory profile with marked elevation in cytokines and autoimmune-like inflammation, even in those receiving treatment, emphasising the complexity of immune imbalances in GD. There were also notable changes in markers of mitochondrial dysfunction, such as elevated oxidative stress markers and altered acylcarnitine profiles, which suggest mitochondrial membrane dysfunction and a reduced capacity for carnitine transport. Additionally, factors such as platelet count, splenectomy, treatment and clinical characteristics were associated with specific omics features, providing further insight into the clinical heterogeneity of GD and potential diagnostic biomarkers.

The exact mechanisms of mitochondrial dysfunction in GD are yet to be fully understood. However, changes in mitochondrial function within GD-affected cells have been observed. Experimental disease models have shown impaired autophagy and proteasomal degradation pathways, as well as an accumulation of dysfunctional mitochondria [39]. In addition, decreased ADP phosphorylation, reduced mitochondrial membrane potential, an increased generation of ROS and free radical damage following the inhibition of the GCase enzyme have been reported in cellular models [40].

Additionally, Ca^2+^ dysregulation in *GBA1* knockout neuronal cells is the driving mechanism for neurodegeneration in GD patients [41]. This Ca^2+^ dysregulation was shown to be consequent of a reduction in mitochondrial Ca^2+^ uptake and reduced expression of the mitochondrial calcium uniporter. It leads to a collapse in the mitochondrial membrane potential, as well as an irreversible fall in the ATP/ADP ratio.

## 4. Mechanism of Mitochondrial Dysfunction in GD

### 4.1. Mouse Models/Experimental Studies

**Figure 2:**
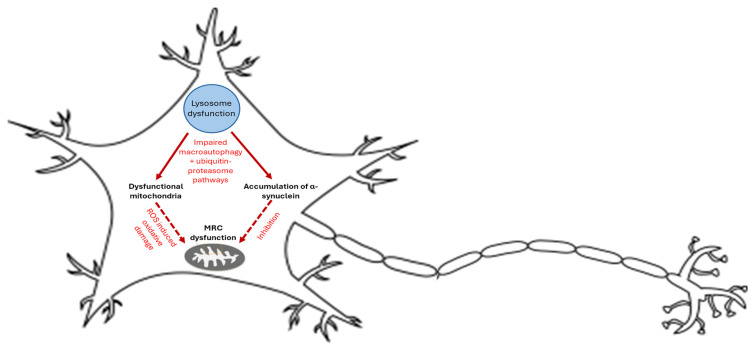
The proposed and putative mechanisms of MRC dysfunction in GD that may contribute to neurodegeneration. The above figure illustrates the potential mechanisms linked to the progression of neurodegeneration in patients carrying the mutated *GBA1* gene. Broken arrows indicate speculated mechanisms.

In the gba mouse model of GD type II, evidence of impaired macroautophagy and a defective ubiquitin–proteasome system involved in the degradation of damaged and misfolded proteins was reported [39]. The impairment of these degradation pathways has been associated with the cerebral accumulation of dysfunctional mitochondria and protein aggregates including the neural protein, α-synuclein, together with the compounds glucocerebroside and GlcSph, which can be toxic to neurons at high levels [29,39]. In addition, α-synuclein has been reported to be able to directly impair MRC complex I activity under in vitro conditions as the result of either direct inhibition or indirect perturbation of mitochondrial membrane lipids (Figure 2) [42], although this has yet to be confirmed in GD animal model and patient studies. Aggregated dysfunctional and fragmented mitochondria are normally removed from the cell in the process of mitophagy. In the mouse model of GD type II, dysfunctional mitochondria were not found to be marked for turnover by the PINK 1-Parkin mitophagy pathway and thus accumulated within the cytosol of the neural cells [39]. As a result of severely impaired MRC function, the mitochondrial membrane receptor PINK1 cannot be imported into the inner mitochondrial membrane; thus, it resides on the outer mitochondrial membrane, where it recruits the cytosolic E3-ubiquitin ligase Parkin, which then initiates mitophagy [39]. In contrast, however, the MRC dysfunction (decreased complex I and II-III activities) in neural cells from the mouse model appeared to result in insufficient dissipation of mitochondrial membrane potential required for PARKIN recruitment [39]. This may have been due to a reversal of MRC complex V activity, maintaining some degree of the membrane potential which has been reported in other cell models of mitochondrial disease, although this can only be speculated at the moment and more studies are required to confirm or refute this potential mechanism [39]. This phenomenon, together with aberrations in the macroautophagy pathway, may have prevented the removal of dysfunctional mitochondria from the cell [39]. Finally, the accumulation of dysfunctional mitochondria with impaired MRC activity may result in a concomitant increase in ROS generation, which may then cause a further loss in MRC activity due to oxidative stress-induced impairment of the enzyme, inner mitochondrial membrane or mitochondrial DNA [39]. However, this can only be speculated at the present time and requires confirmation. Figure 2 outlines the proposed and putative mechanism of mitochondrial dysfunction in GD based on the findings of the study by Osellame et al., 2013 [39]. In common with other organelle membranes, the lysosomal membrane is susceptible to oxidative damage caused by ROS, which may result in further impairment of the lysosome due to the rupture of the organelle’s membrane and the release of hydrolytic enzymes into the cytosol, leading to the proteolytic degradation of cellular systems and organelles, further exacerbating disease pathophysiology [43]. It is important to stress there is a paucity of information linking GD with secondary mitochondrial dysfunction, and further studies are required to confirm this relationship in more detail.

### 4.2. Human Studies

Apart from cerebral mitochondrial dysfunction, evidence of impaired MRC function and decreased co-enzyme Q10 (CoQ10) status has been reported in fibroblasts from patients with GD type III in association with increased mitochondrial ROS generation [44]. Although further studies are required for confirmation, mitochondrial dysfunction in GD appears to be caused by a deficiency in GCase activity. This is given further credence by the presence of GCase in the mitochondria, where it is thought to maintain the integrity and function of the MRC complex I [45]. However, once the mitochondria are impaired, they will impact upon lysosomal function and consequently GCase activity as the result of increased ROS and decreased ATP availability, the latter being required to maintain the acidity of the lumen of the organelle [31].

Another experimental study has shown that induced GBA deficiency in cultured human fibroblasts increases the amount of ROS within the cells [46]. Several other studies investigating oxidative stress in GD have suggested that the presence of redox impairment plays a role in the disease’s pathogenesis, although the evidence for this has varied among different studies [44,46,47].

## 5. Clinical Evidence

Several inborn errors of metabolism, including GD, have been associated with increased ROS and antioxidant deficiencies [48,49,50,51].

The available therapies for GD improve many of the symptoms associated with GD, but some patients continue to report pain and fatigue. The study by Kartha et al. [49] has previously investigated oxidative stress with regard to the therapy. The authors observed significantly different concentrations in oxidative stress biomarkers in treatment-naive GD patients compared to healthy control. In patients on established ERT, results were between the controls and the treatment-naive patients. Importantly, even asymptomatic and minimally symptomatic untreated patients had evidence of significant systemic oxidative stress [49].

Persistent symptoms may be attributed to the underlying inflammation associated with the release of pro-inflammatory cytokines and other mediators [49], either directly from Gaucher macrophages or indirectly through interaction between Gaucher cells and immunomodulatory lymphocytes [52]. Moreover, enzymatic deficiency in patients with GD may induce a cascade of events that leads to the production of ROS, resulting in a state of oxidative stress and inflammation [47,53]. Oxidative stress can disturb several signalling pathways and influence multiple biological processes by modifying proteins, promoting inflammation, inducting apoptosis and impairing mitochondrial function, as well as through many other mechanisms. These effects often accelerate pathological progression and exacerbate disease manifestations [54]. It has also been shown that neuro-chemical abnormalities in patients with GD might be related to oxidative stress and inflammation in the brain [44], which leads to neurodegenerative changes.

There is a clinical need for biomarkers to diagnose and monitor these changes in affected symptomatic and asymptomatic patients [30,31]. A study by Adly et al. (2025) [24] describing the use of vitamin E supplementation for the treatment of oxidative stress in GD patients showed that all individuals with GD had low levels of vitamin E, as well as antioxidant enzymes (GSH, SOD, GPX and PRDX2). Researchers also simultaneously observed high levels of lipid peroxidation compared with healthy controls, indicating the presence of a state of oxidative stress among patients with GD [24]. Further research in the lipid peroxidation pathways could provide more information on the potential disease-specific biomarkers.

Several bioactive molecules are strongly suspected to be involved in the pathophysiology of GD, including lyso-GL1 and other markers of immune activation and systemic/CNS inflammation. It is therefore necessary to critically assess the relationship between these bioactive markers and oxidative stress within GD, based on the findings of this review. The significant correlation between vitamin E, PRDX2 and SSI and lyso-GL1 levels may suggest a direct relation between oxidative stress and GD severity, and that ERT can affect oxidative stress [38].

## 6. Advances in the Treatment of GD

In recent years, the biochemical treatment of GD has seen promising advancements, such as chaperone therapy, substrate reduction therapy (SRT) and gene therapy.

### 6.1. Pharmacological Chaperone Therapy

**Figure 3:**
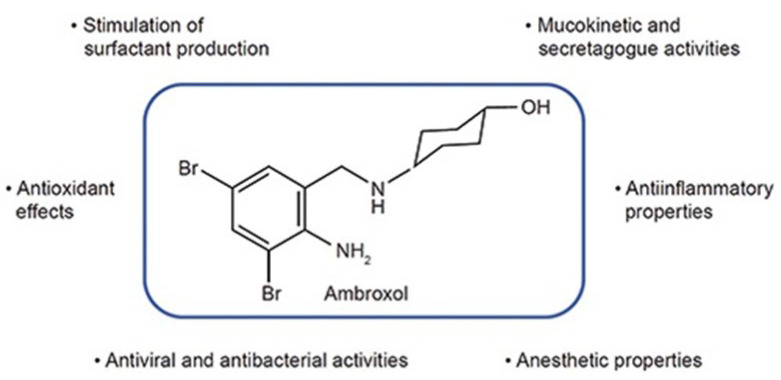
Structure and properties of Ambroxol [55]. Ambroxol can increase the enzymatic activity and lysosomal fraction of mutant GCase variants derived from skin fibroblasts from Type 1 and 2 GD patients [56].

Chaperone therapy can aid correct folding of mutant GCase molecules in the endoplasmic reticulum (ER), assisting trafficking to the lysosomes. Ambroxol, a drug marketed as an expectorant, is an example of a compound which may function as a chaperone capable of facilitating this process for GCase (Figure 3).

This therapy could have a potentially significant use in treating neuronopathic forms of GD due to the increase in lysosomal GCase protein and enzymatic activity. Current research has shown that response to chaperone therapy can be patient-specific and, therefore, identification of a wider range of pharmacological chaperones for mutant GCase variants is a required point of further research [56].

### 6.2. Substrate Reduction Therapy

**Figure 4:**
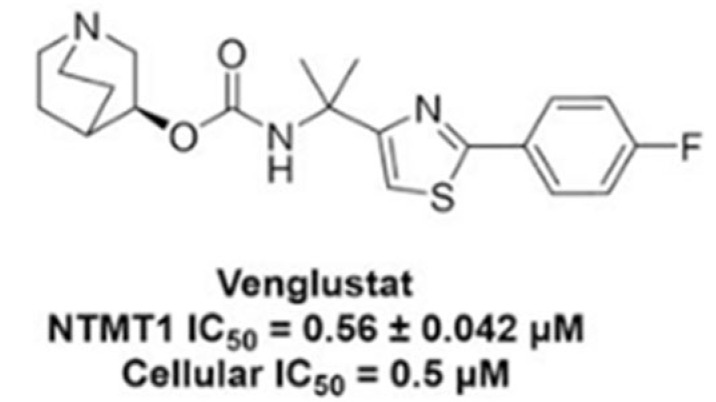
Structure of Venglustat [58]. Venglustat itself is a small-molecule GlcCer synthase inhibitor which can reduce GC production and therefore has use in treatment of GD [59].

SRT is an oral treatment for GD which aims to restore metabolic homeostasis by reducing the biosynthesis of glucocerebroside, which can ameliorate the accumulation of cognate metabolites and glycolipids [57]. The experimental study by Peng et al. [57] has shown that the use of GZ452, which is an analogue of venglustat, can normalise the excess accumulation of substrates such as GlcCer and GlcSph, which cause disruption of cellular functions in mouse models of neuronopathic GD (Figure 4).

This study has also shown that use of GZ452 can also normalise levels of transcription factor EB whilst improving oxygen consumption rate, restoring mitochondrial membrane potential to normal levels and reducing GlcCer levels in mitochondria. In addition, GZ452 normalised GlcCer levels and inhibited GlcCer production in GD neurons, which led to improved neuronal growth due to enhanced mitochondrial function, restored autophagy and reduced mammalian target of rapamycin complex 1 hyperactivity [57]. In contrast, the double-blind clinical trial (NCT02906020) did not demonstrate any advantageous effects when compared to a placebo [59].

SRT has also been shown to be effective in treating type 3 GD by use of eliglustat, which halted the progression of platelet count decline and was associated with serum angiotensin-converting enzyme level improvement; however, this improvement was only seen in one patient, and therefore further investigation would be required [60].

### 6.3. Gene Therapy

Gene therapy is the technique of a therapeutic gene delivery through a vector to targeted cells, and the introduction of a healthy gene copy which can replace diseased copies. Non-viral gene therapy has been seen to have limited efficiency and poor selectivity, and therefore viral gene therapy appears to be a more suitable treatment for therapeutic and clinical application [61,62]. There are some active clinical trials for viral gene therapy of GD, such as the GALILEO-1 trial (Table 2), which aims to treat GD type-1 with FLT201, a liver-directed adeno-associated virus (AAV) capsid which has so far shown an uptake of GCase cells by the bone marrow, spleen and lung [62].

There are many promising potential biochemical treatments for GD which look to improve patient care and outcome. More rigorous pre-clinical trials are necessary to establish the efficacy and safety of treatment methods such as AAVs; however, the research into treatment of the different subtypes of GD is promising at this moment in time.

## 7. Adjunct Therapies

In view of the reported secondary mitochondrial dysfunction and oxidative stress associated with GD [55,71], the efficacy of certain adjunct therapies that target these parameters have been evaluated in this disorder. A study by De la Mata and colleagues in 2015 [44] reported that mitochondrial function and GCase activity were partially restored in GD patient fibroblasts treated with pharmacological chaperone therapy together with CoQ10 supplementation. CoQ10 treatment was able to increase GCase activity and improve mitochondrial function [44]. CoQ10 serves as an essential electron carrier within the MRC and as a potent fat-soluble antioxidant [54], and therefore the beneficial effects of CoQ10 treatment in GD may be due to its ability to improve residual MRC activity as well as ameliorate oxidative stress in patient cells [54]. A more recent study by the same group utilising chemically induced GD macrophages reported the ability of CoQ10 supplementation to decrease lysosomal pH, improve autophagy flux and mitochondrial function (as indicted by an increase in mitochondrial membrane potential), decrease cellular oxidative stress and reduce inflammasome activation [72]. As well as its role as an electron carrier and antioxidant, CoQ10 is also able to directly modulate the action of genes involved in inflammation and may control the release of pro-inflammatory cytokines [7]. The ability of CoQ10 to enhance lysosomal acidification as reported in the study by de la Mata et al. (2017) [54] may reflect its ability to enhance MRC function, which can then provide ATP for the lysosomal ATPase, which pumps protons into the lumen of the organelle, or its role as an electron carrier in the lysosomal electron transport chain that also contributes to maintaining lysosomal acidity [7]. GCase, together with the other lysosomal hydrolases, functions optimally in the pH range, 4–5.1 [7], and therefore, the secondary mitochondrial dysfunction associated with GD may cause a loss of lysosomal pH.

With that, use of the synthetic aromatic cationic tetrapeptide Elamipretide may be judicious in the treatment of GD, since this agent has been reported to increase both mitochondrial respiration and mitochondrial biogenesis in both cell and animal studies, as well decreasing oxidative stress, and has the potential to cross the blood–brain barrier (BBB) and target cerebral mitochondrial dysfunction [24]. The enhancement of TFEB (master gene for regulating lysosomal biogenesis) expression has the potential to boost mitophagy by maintenance of the autophagy–lysosome pathway, in addition to its ability to augment mitochondrial biogenesis. Indeed, targeted dephosphorylation of this transcription factor has been reported to induce nuclear translocation and some transcriptional activity of TFEB [36]. The clinical applications and outcomes of the potential adjunct therapies are outlined in Table 3.

Evidence of systematic oxidative stress has been reported in GD patients (children, adolescents and adults), indicated by increased circulatory levels of the stable lipid peroxidation product, malondialdehyde (MDA), and decreased levels of the antioxidants/antioxidant enzymes: glutathione (GSH), vitamin E, catalase, glutathione peroxidase and peroxiredoxin 2 [50,57]. Vitamin E and peroxiredoxin 2 levels were found to have a negative correlation with the disease severity score index (SSI) [36]. Following 6-month treatment of GD patients with vitamin E, a decrease in oxidative stress, together with a reduction in SSI and liver and spleen volumes, was observed. However, no neurological improvement was reported [36], which may reflect the short duration of the trial or the inability of vitamin E to cross the BBB [36]. In view of the evidence of decreased GSH status in GD patients’, therapeutic candidates such as EPI-743 may be appropriate to restore the levels of this tripeptide antioxidant. EPI-743 can cross the BBB and restore cellular levels of GSH and has shown efficacy in the treatment of primary mitochondrial disease patients [72].

Mitochondrial GCase fosters the MRC complex I function measured as the ratio of NADH oxidase/co-enzyme Q reductase activities [37,81]. Further research by Szego et al. [79] has suggested that another chaperonin protein, HSP10, may impact the mitochondrial function by becoming sequestered by α-synuclein in the cytosol.

## 8. Limitations

The preponderance of evidence about mitochondrial dysfunction in GD comes primarily from experimental (mouse model and in vitro) studies, with limited human validation. Furthermore, the available clinical evidence is limited in view of the heterogeneity in patient populations and study designs, and this may contribute to the inconsistencies between the results of experimental studies and the clinical evidence available. There is a lack of long-term outcome data for current interventions as well as proposed adjunct therapies. In addition, there may be a potential publication bias that favours the results of positive findings, limiting the availability of studies which show no evidence of mitochondrial function in GD, together with those that show minimal or no response to treatment.

## 9. Conclusions

Oxidative stress is related to disease severity in GD patients. The currently available therapies do not cross the BBB and although they can attenuate, they do not fully resolve chronic oxidative stress. Small-molecule therapy and gene therapy target the CNS, but at this stage it is unknown to what extent they will improve mitochondrial function. The proposed adjunct therapies that have been outlined in this review require rigorous clinical trials to establish safety and efficacy before their clinical implementation There is a clinical need for better diagnostic and disease-monitoring biomarkers of mitochondrial dysfunction and oxidative stress in GD, and these would also serve as potential targets for the development of new therapies. However, at present there are only a limited number of studies that have identified evidence of mitochondrial dysfunction in GD, and the majority of this evidence has come from animal models of the disease. Further studies are required to investigate the true preponderance of secondary mitochondrial dysfunction in GD patients.

## Figures and Tables

**Table 1:** Table adapted from d’Amore et al. [8]. The table shows the clinical classification of the three different types of GD. The categories for GD type are not distinct, as patients can have a spectrum of symptoms, along a phenotypic continuum. These manifestations range from mild to severe, including the neurological effects. Type 3 can also be subdivided into three further subgroups: 3a, 3b and 3c [9].

	Non-Neuronopathic (Type 1)	Acute-Neuronopathic (Type 2)	Chronic-Neuronopathic (Type 3)
**Prevalence**	1:40,000–1:60,000	<1:100,000	<1:50,000–1:100,000
**CNS involvement**	None	Severe	Mild to severe; (progressive)
**Clinical manifestations**	Skeletal abnormalities; anaemia; thrombocytopaenia; hepatosplenomegaly (all progressive)	Supranuclear gaze palsy (SNGP); hydrops fetalis; ichthyosis [10]; hepatosplenomegaly (moderate); thrombocytopaenia (severe).	Skeletal abnormalities; anaemia; thrombocytopaenia; hepatosplenomegaly (all progressive)
**Neurological symptoms**	-	Lethal neurological impairment; strabismus opisthotonus, trismus.	Abnormal eye movements (i.e., VIth nerva palsy); ataxia; seizures; dementia (usually appearing later in life) [11]
**Age of onset**	Any age [12]	Infancy [9]	Childhood to adolescence [10]
**Life expectancy**	>60 years [13]	Up to 2 years [14]	~30–40 years [15], but clinical experience shows the clinical experience is much longer (up to the seventh decade) in patients with milder clinical phenotypes.

**Table 2:** Therapies in GD—mechanisms of action and impact on the CNS function.

Drug	Mechanism of Action	Reference	Trial Number	CNS Target Organ Y/N
**Crossing BBB**				
Ambroxol (clinical trial)	Iminosugar, GCase chaperone; binds to GCase, facilitating its trafficking to the lysosome, and in a low pH it is released, increasing GCase activity in cultured macrophages derived from GD and GBA1-PD patients by ∼3.5-fold, while reducing substrate levels by ∼2-fold when compared to untreated cells; in neuronopathic GD patients with N188S, G193W, F213I/RecNciI and D409H/IVS10-1G > A genotypes	[63,65] [66,67]	NCT02941822, NCT05778617, NCT05830396, NCT05287503, NCT02914366, NCT04388969, NCT0458825	Y [38]
Gene therapy (Clinical trial)	Augmentation of residual GCase expression, and its potential to improve the clinical phenotype by reduction and prevention of cellular accumulation of GCase substrate.	-	NCT05324943	Y
GZ452 (Venglustat analogue) (Mouse model) Venglustat (clinical trial)	SRT	[58] [59]	NCT02906020 NCT02843035	Inhibited GlcCer production and normalised GlcCer levels in GD neurons Y, but no significant reduction in GlcCer in the CNS of GD patients
**Not crossing BBB**				
SRT: Eliglustat Miglustat (clinical trial)	Inhibiting UDP-GlcCer synthase, an enzyme that catalyses GlcCer biosynthesis, reducing GlcCer influx load into the lysosome. Inhibitors of GlcCer synthase.	[68]	- -	N N
ERTs: Imiglycarase Veraglucerase alpha Taliglucerase alpha And their Biosimilars (clinical trial)	Delivers a functional enzyme to break down the accumulated GlcCer. Reduces hepatosplenomegaly, improves anaemia and thrombocytopenia and ameliorates skeletal damage like bone pain and crises.	[64] [69]	-	N N

**Table 3:** Adjunct therapies in GD patients—clinical application and outcomes.

Drug	Mechanism of Action	[Reference] or NCT	Study Population	Outcome Measures/CV%	Effect Size	Biochemical Parameters and Safety Considerations
Co-enzyme Q10 (experiemental studies)	Reduce GlcCer accumulation, mitochondrial dysfunction and oxidative stress in chemically induced GD macrophages model	[7,39,44]	-	-	-	The safety of supplemental Co-enzyme Q10 is well established in more than 200 randomised controlled clinical trials in a wide range of disorders, with CoQ10 at doses of 200–300 mg/day for 3–6 months typically utilised, although some studies have used much higher daily doses (2700 mg/day) No serious adverse effects were reported [73].
Inhibitors of Ca^2+^ channels, such as diltiazem and verapamil (experimental studies)	By increasing ER calcium concentrations and activity of Ca^2+^-dependent endogenous molecular chaperones, were able to partially restore lysosomal enzyme folding, trafficking and activity	[70,74]	-	-	-	The potential side effects of calcium channel blockers were assessed in the systematic review by Hatamian et al., 2025 [75] which concluded that the effectiveness of these drugs many vary depending on the dosage and patient population.
Vitamin E (tocopherol) (clinical trial) EPI-743	Targets oxidoreductase enzymes essential for redox control of metabolism	[24]-NCT06211478 [76] [72]	6 GD1 14 GD3	SSI decreased from 9.5 (8–13) to 5.5 (3–10) lyso GL1 decreased from 178 (120–208) ng/mL to 146.7 (89.8–184.6) ng/mL SSI decreased from 14 (11–16) to 8.5 (7–11) lyso GL1 decreased from 247.55 (179.8–473.0) ng/mL to 203.5 (152–301) ng/mL	*p* = 0.017 *p* = 0.001 *p* < 0.001	MDA and antioxidant markers including peroxiredoxin 2, Glutathione peroxidase, superoxide dismutase and reduced glutathione. High-dose vitamin E supplementation may affect normal cellular processes including immunity and cell growth, contribute to oxidative stress and also amplify the risk of bleeding [77]. EPI-743 treatment has been demonstrated to be safe and well tolerated [78].
HSP10 (Experiemental study)	Chaperonin protein	[79]	-	-	-	Mitochondrial GCase promotes the maintenance of mitochondrial complex I measured as the ratio of NADH oxidase/co-enzyme Q reductase activities. More clinical studies are required to assess the potential side effects associated with HSP10.
*N* -acetylcysteine (NAC; 7200 mg/day) (clinical trials)	Anti-inflammatory properties	NCT02583672 NCT02437396	20 subjects	-	-	Concentration of glutathione in brain (μmol/g) after months, measured by MRI. Complement 5A and Hepcidin NAC is well tolerated in oral doses below 1200 mg/day and has anticoagulant and platelet-inhibiting properties [80].

## Data Availability

No new data were created or analysed in this study. Data sharing is not applicable to this article.

## Supplementary Materials

The following supporting information can be downloaded at: https://www.mdpi.com/article/10.3390/genes16111269/s1, File S1: Methodology: Literature search.

## Abbreviations

The following abbreviations are used in this manuscript:

ATP	Adenosine Triphosphate
BBB	Blood–brain barrier
CNS	Central nervous system
GCase	Glucocerebrosidase
GlcCer	Glucosylceramide
GlcSph	Glucosylsphingosine
ETC	Electron transport chain
ER	Endothelial reticulum
ERT	Enzyme replacement therapy
GD	Gaucher disease
LSD	Lysosomal storage disorder
MRC	Mitochondrial respiratory chain
ROS	Reactive oxygen species
SNGP	Supranuclear gaze palsy
PD	Parkinson disease
SRT	Substrate reduction therapy
SSI	Severity score index
