# Supplementary material for: Secondary Mitochondrial Dysfunction in Gaucher Disease Type I, II and III—Review of the Experimental and Clinical Evidence
Source: Genes (Basel). 2025 Oct 28;16(11):1269. doi: 10.3390/genes16111269 (PMC12652696; doi:10.3390/genes16111269)
Supplement: Supplementary file 1 [file genes-16-01269-s001.zip › genes-3887704-supplementary.pdf]

Methodology: Literature search

Seach terms:

The search was performed using combinations of several keywords: 'Gaucher disease', 'oxidative stress', 'antioxidants', 'vitamin E', 'tocopherol', 'clinical trials', 'neurodegeneration', 'lysosomal storage disease', 'central nervous system', 'ROS', 'mitochondrial', 'mitochondrial dysfunction', 'clinical outcome', 'mortality' with Boolean operators "AND" and "OR" for a thorough search. We performed a descriptive narrative synthesis.

Search strategy:

Gaucher disease AND mitochondria	65 articles
Lysosomal storage disease AND mitochondria	395 articles
Lysosomal storage disease AND oxidative stress AND Gaucher	34 articles
Gaucher AND/OR secondary mitochondrial dysfunction	6 articles
Gaucher AND antioxidants	83 articles
Gaucher AND oxidative stress AND mitochondria	14 articles
Gaucher AND oxidative stress AND neurodegeneration	5 articles
Gaucher AND tocopherol	2 articles
Gaucher AND/OR ROS	21 articles
Gaucher AND mitochondria AND clinical outcome	0 articles

The overall number of articles was: 625

Duplications excluded: 157

Reviewed: 468 articles

Inclusion criteria: articles relevant to experimental, cellular and animal models, clinical cases and clinical trials

Excluded: reviews and papers in a non- English language, conference abstracts

Conflict resolution: a third reviewer was invited to comment on the paper and whether it is worth including it
